# Cancer Testis Antigens and Immunotherapy: Expression of PRAME Is Associated with Prognosis in Soft Tissue Sarcoma

**DOI:** 10.3390/cancers12123612

**Published:** 2020-12-03

**Authors:** Markus Albertsmeier, Annelore Altendorf-Hofmann, Lars H. Lindner, Rolf D. Issels, Eric Kampmann, Hans-Roland Dürr, Gabriele Schubert-Fritschle, Martin K. Angele, Thomas Kirchner, Achim A. Jungbluth, Thomas Knösel

**Affiliations:** 1 Department of General, Visceral and Transplantation Surgery, University Hospital, Ludwig-Maximilians-Universität (LMU) Munich, Marchioninistr. 15, 81377 Munich, Germany; martin.angele@med.uni-muenchen.de; 2 Department of General, Visceral and Vascular Surgery, Friedrich-Schiller Universität Jena, Am Klinikum 1, 07743 Jena, Germany; annelore.altendorf-hofmann@gmx.de; 3 Department of Internal Medicine III, University Hospital, Ludwig-Maximilians-Universität (LMU) Munich, Marchioninistr. 15, 81377 Munich, Germany; Lars.Lindner@med.uni-muenchen.de (L.H.L.); rolf.issels@med.uni-muenchen.de (R.D.I.); kampmann77@gmail.com (E.K.); 4 Musculoskeletal Oncology, Department of Orthopaedic Surgery, Physical Medicine and Rehabilitation, University Hospital, Ludwig-Maximilians-Universität (LMU) Munich, Marchioninistr. 15, 81377 Munich, Germany; hans_roland.duerr@med.uni-muenchen.de; 5 Munich Cancer Registry (MCR) of the Munich Tumour Centre (TZM), Institute for Medical Information Processing, Biometry, and Epidemiology (IBE), University Hospital, Ludwig-Maximilians-Universität (LMU) Munich, Marchioninistr. 15, 81377 Munich, Germany; gabriele.schubert-fritschle@med.uni-muenchen.de; 6 Institute of Pathology, Ludwig-Maximilians-Universität (LMU) Munich, Thalkirchner Str. 36, 80337 Munich, Germany; Thomas.Kirchner@med.uni-muenchen.de; 7 Department of Pathology, Memorial Sloan Kettering Cancer Center (MSKCC), New York, NY 1275, USA; jungblua@mskcc.org

**Keywords:** soft tissue sarcoma, human, cancer/testis antigens, PRAME, NY-ESO-1, SSX2, biomarker, tumour infiltrating lymphocytes, immunohistochemistry

## Abstract

**Simple Summary:**

Soft tissue sarcomas are a group of malignant tumors developing from connective tissues in different parts of the human body. In the face of limited options for systemic therapy, immunotherapeutic approaches are currently being explored. In this context, cancer testis antigens (CTAs) are potential targets for cancer immunotherapy due to tumor-specific patterns of expression and high immunogenicity. We, therefore, aimed to describe the expression of three CTAs, PRAME, NY-ESO-1, and SSX2, and analyze their prognostic value in a large cohort of high-risk soft tissue sarcomas with long-term follow-up. Our results show sarcoma subtype-specific patterns of CTA expression and we demonstrate an association of CTAs with overall survival, especially for PRAME. Our results provide support for future trials investigating CTA-directed immunotherapy in eligible patients with various sarcoma subtypes and they may help establish CTAs as diagnostic tools in soft tissue sarcoma.

**Abstract:**

(1) Background: PRAME, NY-ESO-1, and SSX2 are cancer testis antigens (CTAs), which are expressed in testicular germ cells with re-expression in numerous cancer types. Their ability to elicit humoral and cellular immune responses have rendered them promising targets for cancer immunotherapy, but they have never been studied in a large and well-characterised cohort of soft tissue sarcomas (STS). (2) Methods: On a protein level, we examined PRAME, NY-ESO-1, and SSX2 expression in tumour tissues of 249 high-risk STS using immunohistochemistry. We correlated expression levels with clinicopathological parameters including tumour-infiltrating lymphocyte (TIL) counts, grading, and long-term survival. (3) Results: Expression of PRAME, NY-ESO-1, and SSX2 was observed in 25 (10%), 19 (8%), and 11 (4%) of 249 specimens with distinct patterns for histo-subtypes. Expression of PRAME was associated with shorter patient survival (*p* = 0.005) and higher grade (G2 vs. G3, *p* = 0.001), while NY-ESO-1 expression was correlated with more favourable survival (*p* = 0.037) and lower grade (G2 vs. G3, *p* = 0.029). Both PRAME and NY-ESO-1 expression were more frequent in STS with low TIL counts. In multivariate analysis, high PRAME and low SSX2 expression levels as well as metastatic disease and non-radical resections were independent predictors of shorter overall survival. (4) Conclusions: CTAs PRAME, NY-ESO-1, and SSX2 show distinct expression patterns in different STS subtypes. These results demonstrate their prognostic relevance and may guide future immunotherapeutic approaches in STS.

## 1. Introduction

Soft tissue sarcomas (STS) are a heterogenous group of rare malignant tumours originating from soft tissues that can occur in different parts of the human body. While treatment of localised disease is based on radical resection, evidence from a limited number of trials supports the use of perioperative systemic therapy in high-risk tumours [1,2]. Therapeutic options, however, are limited, and in a recent trial, a histology-tailored regimen was inferior to standard chemotherapy with epirubicin and ifosfamide [2]. Immunotherapy, which has been successfully introduced for several cancers, might be an option to individualise therapy of sarcoma patients [3].

In normal tissues, expression of cancer-testis antigens (CTAs) is restricted to germ cells and trophoblasts. They are aberrantly expressed in various types of cancer. Following their identification in malignant melanoma, CTAs have been detected in carcinomas of various sites, such as lung, ovary, urinary bladder, liver, and other organs [4]. Several CTAs, like *MAGE*, NY-ESO-1, and PRAME, are expressed in different sarcoma subtypes, such as synovial sarcoma [5,6,7,8,9,10,11], myxoid/round cell liposarcoma [6,11,12,13,14,15,16], and other soft tissues sarcomas. CTAs can be highly immunogenic and are considered potential targets for immunotherapy of cancer [17].

Tumour-infiltrating lymphocytes (TILs) belong to the microenvironment of malignant tumours and constitute an essential part of the human body’s dynamically changing reaction to the tumour. While some TIL populations have been shown to promote tumour progression [18], others are implicated in killing tumour cells or enhancing the tumour response to certain chemotherapeutics [19]. Although high TIL counts following neoadjuvant treatment of high-risk soft tissue sarcomas have been associated with a more favourable prognosis [20], the role of TILs in chemo-naïve STS is less clearly defined [21].

In the present study, we analyse the expression of CTAs NY-ESO-1, PRAME, and SSX2 in a large and well-characterised cohort of high-risk soft-tissue sarcoma patients with long-term follow-up and correlate our findings with the presence of TILs, clinical tumour characteristics, and survival data.

We show that PRAME, NY-ESO-1, and SSX2 display distinct expression patterns in different STS subtypes. PRAME and NY-ESO-1 expression levels are correlated to patient survival and tumour grade in opposing ways, while both CTAs are correlated with low TIL counts. In multivariate analysis, high PRAME and low SSX2 expression levels as well as metastatic disease, non-radical resections, and not receiving chemotherapy are shown to be independent predictors of shorter overall survival. These results may guide future immunotherapeutic approaches in STS.

## 2. Results

### 2.1. Patient Cohort

The study included 249 patients (female *n* = 125, male *n* = 124) with intermediate (*n* = 118) or high-grade (*n* = 131) STS. The patients’ age at the time of diagnosis ranged from 18 to 79 years (median age, 53 years). Parameters describing the tumours of included patients (histological subtype, location, grading, size, and presence of metastatic disease) and their treatment (surgical outcome, radiotherapy, and regional hyperthermia) are shown in Table 1. All patients received anthracycline-based systemic therapy. Metastatic disease, if present, was judged as surgically respectable at initial evaluation. Nearly all patients (*n* = 223, 89.6%) underwent surgical resection, and about one in five patients received additional radiotherapy.

### 2.2. Cancer-Testis Antigens Show Distinct Expression Patterns in Sarcoma Subtypes

**Figure 1:**
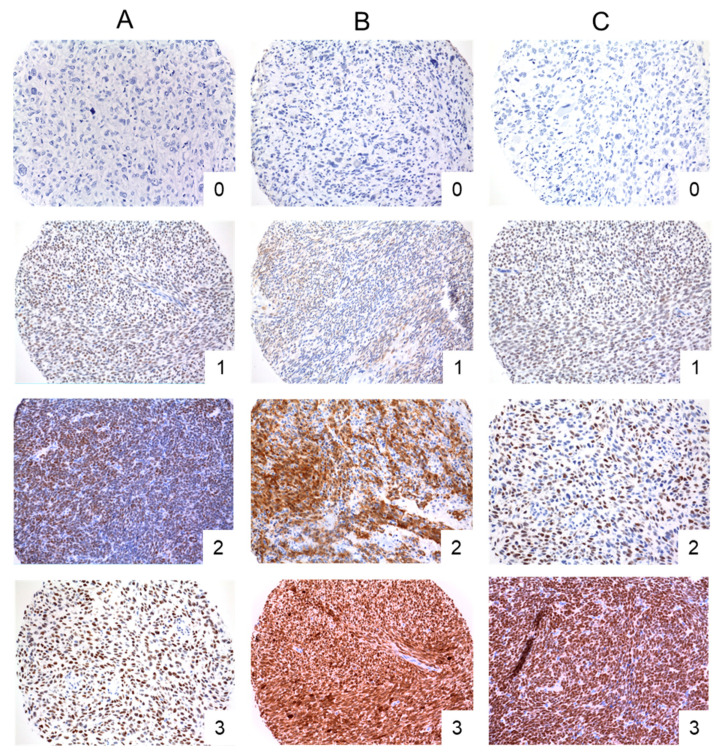
Representative micrographs of cores on a tissue microarray stained for (**A**) PRAME, (**B**) NY-ESO-1, and (**C**) SSX2. Numbers represent semiquantitative scoring of immunostaining: 0, negative; 1, weak; 2, moderate and 3, strongly positive. Magnification 20×.

Expression of PRAME, NY-ESO-1, and SSX2 was observed in 25 (10%), 19 (8%), and 11 (4%) of 249 specimens (248 specimens for SSX2), respectively. Examples of immunohistochemistry staining for CTAs are shown in Figure 1, and detailed results of TMA analysis with respect to histologic subtypes are shown in Table 2. Expression levels for all CTAs are shown in Table A1.

### 2.3. Cancer-Testis Antigens PRAME and NY-ESO-1 Are Expressed More Frequently in Soft Tissue Sarcomas with Low Counts of Tumour-Infiltrating Lymphocytes

**Figure 2:**
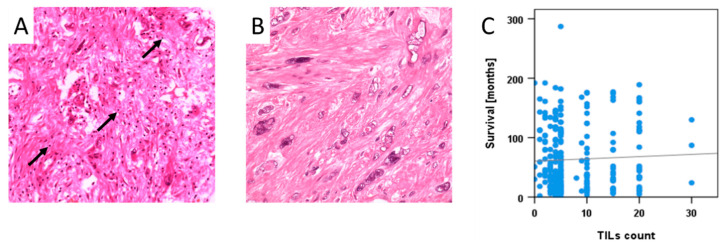
(**A**) and (**B**): representative samples of UPS (**A**) with >4/HPF tumour-infiltrating lymphocytes (TILs) (“positive”) and (**B**) without TILs (≤3/HPF, “negative”). Pictures are representative micrographs of cores on an H&E stained tissue microarray. Black arrows pointed at TILs. Magnification 20×. (**C**): Relationship between counts of tumour-infiltrating lymphocytes (TILs) and survival. No significant correlation was found (R^2^ = 0.0002; *p* > 0.05).

Tumour-infiltrating lymphocytes (TILs) were found in 193 (78%) of 249 specimens. Examples of H&E staining of TILs are depicted in Figure 2A,B, and detailed TIL counts for different histologic subtypes are shown in Table 2. TIL counts were not statistically correlated to patient survival (Figure 2C).

**Figure 3:**
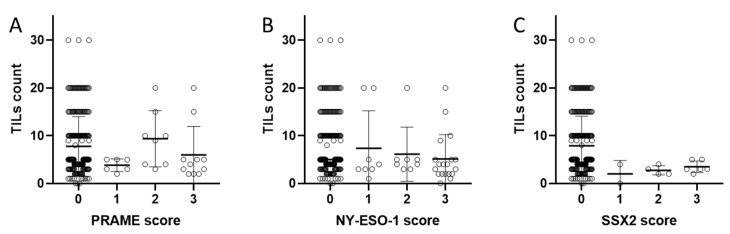
Counts of TILs for different expression levels of (**A**) PRAME, (**B**) NY-ESO-1, and (**C**) SSX2. Numbers represent semiquantitative scoring of immunostaining: 0, negative; 1, weak; 2, moderate; and 3, strongly positive.

**Figure 4:**
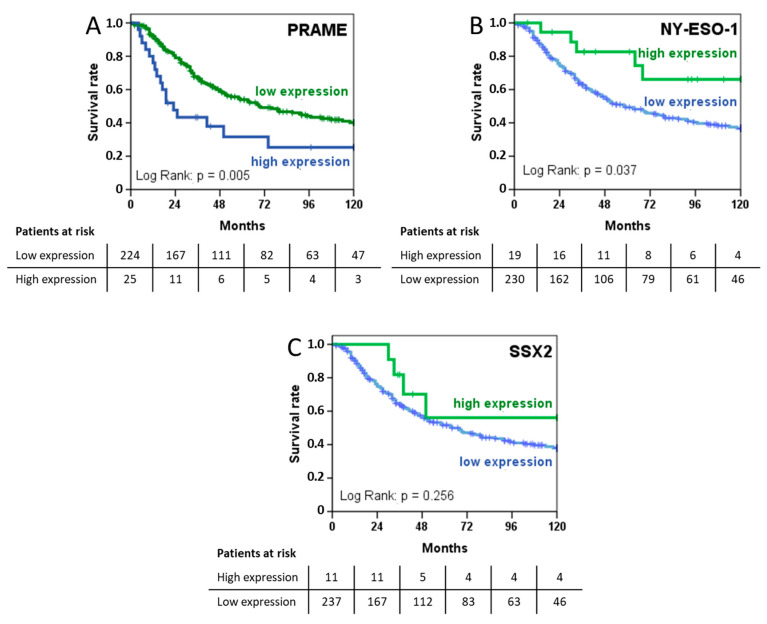
Univariate analysis of overall survival depicted as Kaplan–Meier curves stratified according to the expression of (**A**) PRAME, (**B**) NY-ESO-1, and (**C**) SSX2.

PRAME expression tended to be more frequent in tumours with low numbers of TILs (9/56, 16.1%) than in tumours with high TIL counts (16/193, 8.3%, *p* = 0.126, Figure 3). NY-ESO-1 expression was three times more frequent in tumours with low TIL counts (9/56, 16.1%) than in tumours with high numbers of TILs (10/193, 5.2%, *p* = 0.018, Figure 4). The low frequency of SSX2 expression did not allow for a meaningful statistical analysis.

### 2.4. Expression of PRAME and NY-ESO-1 Is Associated with Grading in Opposing Ways

Expression of PRAME was correlated with higher grading (G2 vs. G3, *p* = 0.001), while NY-ESO-1 expression was seen more frequently in lower grading (G2 vs. G3, *p* = 0.029). The low frequency of SSX2 expression did not allow for a meaningful statistical analysis.

### 2.5. PRAME Expression Is Prognostic of Shorter Survival while NY-ESO-1 Is Associated with a More Favourable Prognosis

Median duration of follow-up was 40 months for all patients, 96 months for 110 patients still alive, and 31 months for 139 patients who had died. An analysis of overall survival using the Kaplan–Meier method is shown in Figure 4. Patients with PRAME-positive tumours had statistically significant shorter median survival (23.0 (13.5–32.5) months vs. 69.0 (44.4–93.6) months, *p* = 0.005), while high NY-ESO-1 expression was associated with improved survival (>120 months vs. 58.0 (39.4–76.6) months, *p* = 0.037). The expression of SSX2 had no significant impact on overall survival. The results of univariate survival analysis for CTAs and clinical parameters are shown in Table A2.

### 2.6. Expression of CTAs PRAME und SSX2 As Well As Radical Resections, Chemotherapy, and Metastatic Disease Influenced Overall Surivival in Multivariate Analysis

To control for confounding factors, a Cox proportional hazards model for overall survival was calculated including the expression of CTAs, treatment variables, and tumour characteristics in a stepwise procedure (Table 3). High PRAME expression levels (HR 2.675 (1.548–4.622), *p* < 0.001) and low expression of SSX2 (HR 3.054 (1.060–8.796), *p* = 0.039) were independent predictors of shorter overall survival. Furthermore, radical resection and the absence of metastatic disease were associated with favourable survival (Table 3). The expression of NY-ESO-1, histological subtype, the use of radiotherapy, or regional hyperthermia and TIL counts did not contribute significantly to the risk of death and, therefore, did not enter the final model.

## 3. Discussion

In this study, we analysed the expression of CTAs PRAME, NY-ESO-1, and SSX2 as well as the presence of tumour-infiltrating lymphocytes (TILs) in a well-characterised cohort of high-grade soft tissue sarcoma patients. Although several CTAs have been studied extensively in the past, little is known about PRAME as well as SSX2 on the in-situ protein expression level. Both antigens were chosen because of the lack of data pertaining their presence in sarcoma. NY-ESO-1 was chosen to serve as a reference because it is among the well-studied CT antigens.

We found the expression of PRAME and the presence of a high number of TILs to be associated with shorter survival, while NY-ESO-1 expression was more frequent in patients with a more favourable prognosis. Furthermore, the expression of PRAME was associated with higher grade and a lower number of TILs while NY-ESO-1 expression was correlated with lower grade and low TILs. Finally, high PRAME expression was confirmed as a prognostic factor for overall survival in a Cox proportional hazards model that included clinical parameters like metastatic disease and surgical margins. To our knowledge, the present study of 249 patient cases is the largest study of CTAs in soft tissue sarcoma demonstrating a prognostic significance of these markers.

Significant PRAME overexpression has been described in uterine carcinosarcoma, synovial sarcoma, and multifocal leiomyosarcoma, while other sarcoma subtypes appear to express PRAME less frequently [22]. In the present study, we found PRAME expression in undifferentiated pleomorphic sarcoma (UPS), malignant peripheral nerve sheath tumour (MPNST), synovial sarcoma, leiomyosarcoma (LMS), angiosarcoma, dedifferentiated liposarcoma (DDLPS), uterine carcinosarcoma, and rhabdomyosarcoma, making PRAME a potential target for immunotherapy in these histological subtypes. Moreover, in our mixed cohort of intermediate and high-grade sarcomas, PRAME expression was prognostic for unfavourable survival, which was not the case in the studies of liposarcoma, leiomyosarcoma, and synovial sarcoma subgroups by others [9,22]. Our results, therefore, might help establish PRAME both as a prognostic marker that can be used in nomograms and as a valuable target antigen in soft tissue sarcoma. The fact that we were able to demonstrate PRAME expression in only 3 of 28 synovial sarcomas compared to 100% overexpression in a gene expression study by Roszik et al. [22] might be due to different methods, detection of PRAME on protein level, and scoring evaluation. The potential of PRAME as a target for immunotherapy may, therefore, be more important than suggested by our results. Future studies should also assess the prognostic value of CTAs in specific histological subtypes.

Currently, PRAME is a target antigen in a clinical phase I trial evaluating multi-tumour-associated antigen-specific cytotoxic T lymphocytes in rhabdomyosarcoma (NCT02239861, TACTASOM). Given that PRAME expression was negatively correlated with lymphocyte infiltration in our study and that PRAME has been described to downregulate antigen-presentation [22], effective strategies for T-cell recruitment and activation will be needed. These may include a boost of MHC class I expression using, e.g., demethylating drugs and other approaches [23], combinatorial therapy with NK cells [24], or checkpoint inhibitors [25].

Our multivariate regression analysis suggests that the prognostic value of PRAME expression is robust, even in relation to established clinical parameters such as surgical margins or metastatic disease. Nonetheless, while most biopsies were taken before the initiation of neoadjuvant treatment, some patients had received radiotherapy, chemotherapy, and/or hyperthermia before histopathological sampling for this study. Radiotherapy potentially induces gene mutations and alters the expression of CTAs [26], which may have influenced our results.

NY-ESO-1 has been studied extensively in various cancers. We found it to be expressed in almost half of synovial sarcomas and in some cases of UPS, LMS, DDLPS, and angiosarcoma. This essentially confirms the results of previous studies, although expression in synovial sarcoma has been demonstrated to be as high as 76% to 80% [10,27,28]. In our cohort of soft tissue sarcoma patients, NY-ESO-1 expression was associated with lower grade, and it was prognostic of more favourable survival, as has been shown in a series of high-grade soft tissues sarcoma by Kakimoto et al. [27]. Interestingly, in non-small-cell lung cancer, high NY-ESO-1 expression was associated with poor prognosis [29], while in epithelial ovarian cancer [30] and breast cancer [31] no relationship with survival has been found. These discrepancies may be caused by the variability of specific interactions between different NY-ESO-1-expressing tumours and their microenvironment, including altered expression of tumour antigens, epitope spreading, and the induction of immunosuppressive cells [32]. Furthermore, NY-ESO-1 is highly immunogenic, and expression might stimulate a T-cell response in some tumours.

Widespread expression across various cancer types has made NY-ESO-1 an attractive target for different immunotherapeutic strategies such as adoptive cell transfer [33,34], cancer vaccines (NCT01883518), and immune checkpoint inhibition [35]. While its strong immunogenic nature implies that therapies directed against NY-ESO-1 may also boost the natural immune response, its restricted expression in normal tissue presumably limits off-target toxicities [32].

Interestingly, in our study, NY-ESO-1 and PRAME were expressed more often in tumours with low TILs. The suppression of CD3^+^ T-lymphocytes has been described in malignant-melanoma-expressing NY-ESO-1, although its mechanism remains unclear [36]. In a previous analysis of our cohort, the expression of PD-L1 on STS was associated with an infiltration by PD-1-positive TILs, high tumour grading, and short survival [37]. These results point to an actual PD-L1 interaction with PD-1-positive TILs in STS and support checkpoint inhibitor therapy in eligible patients. While lymphocyte infiltration is a prerequisite for this approach, it appears that non-T-cell inflamed tumours, which are resistant to PD-1/PD-L1 inhibitors and which were more frequent among NY-ESO-1 and PRAME-positive STS in the present study, can still be treated with adoptive T-cell based immunotherapy [38]. Targeting CTAs may, therefore, provide a therapeutic option in patients not eligible for checkpoint inhibitor therapy.

Although high SSX2 expression was prognostic of favourable survival in multivariate analysis, the low overall frequency of SSX2 expression in the present study did not allow for a meaningful statistical analysis of this CTA in relation to TILs or grading. The SS18-SSX2 fusion, however, was a frequent finding in synovial sarcomas in the analysis of the Cancer Genome Atlas [39]. SSX2 may, therefore, be a target in synovial sarcomas, which were SSX2-positive in 25% in our study and which are treated with SSX2-directed cytotoxic T-lymphocytes in the TACTASOM trial (NCT02239861).

Our study has several limitations. First, no conclusions can be drawn about the prognostic value of CTAs in specific histological subtypes due to the limited number of patient cases. Second, in this specific cohort based on the EORTC-STBSG 62961 trial protocol [40], the majority (78%) of patients received regional hyperthermia, which limits the external validity of our results. Finally, the generalisability of our conclusions is limited by the fact that in 25% of patients, biopsies used in this study had been taken after the initiation of neoadjuvant treatment. Although we have found no indication in our data that systemic therapy influenced CTA expression, results may not be transferable to patients not having undergone neoadjuvant treatment.

## 4. Materials and Methods

### 4.1. Patients

A total of 249 cases of high-risk STS were retrieved from the archives of the Institute of Pathology at Ludwig-Maximilians-Universität (LMU) Munich, Germany. Patients were diagnosed and treated between 1989 and 2012 at the sarcoma centre of LMU University Hospital. Data on clinical parameters were extracted from the original pathology reports and the database of the previously published EORTC-STBSG 62961 trial (NCT00003052) [40]. This included sex; age; primary site; histological subtype; grading; size; presence of metastatic disease; surgical outcome; and whether radiotherapy, chemotherapy, and/or regional hyperthermia had been performed. Patients were followed in outpatient clinics or by contacting their general practitioner, and clinical data were updated until December 2019. The study has been approved by the institutional review board at LMU Munich (No. 20-127).

### 4.2. Histopathology and Tissue Microarray Construction Immunohistochemistry

Biopsies were taken deep from dedifferentiated areas of the tumour before the initiation of neoadjuvant treatment if possible; in 61 patients (25%), only biopsies taken after the initiation of neoadjuvant treatment were available for this analysis. In addition to the original pathology reports, microscopic findings (tumour type according to current WHO classification and degree of differentiation) were reassessed by two authors (E.K. and T.K. (Thomas Knösel)). For tissue microarray (TMA) assembly, representative tumour areas were marked on H&E stained slides of formalin-fixed, paraffin-embedded tumour samples from all patients according to standard procedures [41] and two 0.6 mm punch biopsies were taken of each sample. Samples from tonsils were added as controls.

### 4.3. Immunohistochemistry of Cancer Testis Antigens

Expression of CTAs NY-ESO-1, PRAME, and SSX2 was defined as the primary outcome of this study. Immunohistochemical staining was done on 5 µm TMA sections according to standard procedures. Antibodies against NY-ESO-1, PRAME, and SSX2 were obtained and used for the analysis as detailed in Table 4. All assays were performed on a Leica Bond-3 automated stainer platform (Leica, Buffalo Groves, IL, USA). Before application of the primary, heat-based antigen retrieval was performed employing a high pH buffer (ER2, Leica). A polymeric secondary kit (Refine, Leica) was used to detect the primary. Immunostaining was evaluated and scored semi-quantitatively by two investigators (M.A. and T.K. (Thomas Knösel)) on a four-tier scale: 0, negative; 1, weak; 2, moderate and 3, strongly positive, that was reduced to a 2-tier system (0—low, versus 1/2/3—high for PRAME and SSX2, 0/1/2—low, versus 3, high for NY-ESO-1) for statistical analysis. Five fields were scored for each sample and from duplicate samples; the higher score was used for statistical analysis. In case of discrepancy between the two investigators (*n* = 12), samples were jointly reviewed and a consensus was reached. Researchers scoring the TMAs were blinded to the clinical data.

### 4.4. Tumour-Infiltrating Lymphocytes

Tumour-infiltrating lymphocyte (TIL) counts were defined as a secondary outcome parameter. TILs comprised all mononuclear cells and were counted per high power field (HPF, 400× magnification) in H&E stained TMA slides. Samples with counts of at least four lymphocytes per HPF were considered positive. From duplicate samples, the higher score was used for statistical analysis.

### 4.5. Statistical Analysis

Categorical variables were tested for independence using the Chi square test.

Survival was calculated from the date when sarcoma was first diagnosed. Overall survival (patients’ death without regarding the cause of death) was used as the endpoint for estimating prognosis. Survival curves were created using the Kaplan–Meier method, and the log-rank test was used to assess differences in survival.

Significant and independent predictors of overall survival were identified by Cox proportional hazard analysis. The forward stepwise procedure was set to a threshold of 0.05. All statistical analyses were performed using SPSS 26.0 (IBM, Chicago, IL, USA) software (Figure 3: GraphPad Prism version 8.4.2, GraphPad Software, San Diego, CA, USA).

Statistical significance was defined as a *p* value < 0.05 for all analyses.

## 5. Conclusions

CTAs PRAME, NY-ESO-1, and SSX2 show distinct expression patterns in different STS subtypes. PRAME and SSX2 are prognostic for overall survival and may be used in future nomograms of survival in STS. PRAME and NY-ESO-1 expression levels are correlated to patient survival and tumour grading in opposing ways, while both CTAs are more frequent in tumours with low TIL counts. These results may guide future immunotherapeutic approaches in STS.

## Figures and Tables

**Table 1:** Patient Characteristics. UPS: undifferentiated pleomorphic sarcoma; SFT: solitary fibrous tumour; MPNST: malignant peripheral nerve sheath tumour.

		*n*	%
**Total**		249	100
Sex	Male	124	50
	Female	125	50
Histological subtype	UPS	82	33
	Leiomyosarcoma	50	20
	Synovial sarcoma	28	11
	Dedifferentiated Liposarcoma	47	19
	Angiosarcoma	9	4
	MPNST	13	5
	Other	20	8
Location	Extremities	85	34
	Retroperitoneal	54	22
	Abdominal/visceral	42	17
	Trunk	60	24
	Other	8	3
Grading	Intermediate (G2)	118	47
	High (G3)	131	53
Size	<50 mm	20	8
	50–79 mm	61	24
	80–120 mm	64	26
	>120 mm	76	31
	Missing	28	11
Metastatic disease	M0	227	91
	M1	22	9
Surgical margins	R0/R1	202	81
	R2 or no resection	47	19
Radiotherapy	Done	53	21
	Not done	180	72
	Unknown	16	2
Regional hyperthermia	Done	195	78
	Not done	54	22

**Table 2:** Cancer testis antigen expression and tumour-infiltrating lymphocytes (TILs) according to tumour entity: the table shows the number and percentage of patients with high antigen expression and high TIL infiltration.

	Total	PRAME		NY-ESO-1		SSX2		TILs	
Histologic Subtype	*n*	*n*	%	*n*	%	*n*	%	*n*	%
UPS	82 ^a^	6	7%	2	2%	3	4%	71	87%
Leiomyosarcoma	50	3	6%	1	2%	0	0%	33	66%
Synovial Sarcoma	28	3	11%	12	43%	7	25%	13	46%
DDLPS	47	2	4%	3	6%	0	0%	39	83%
Angiosarcoma	9	3	33%	1	11%	0	0%	8	89%
MPNST	13	5	38%	0	0%	1	8%	11	85%
Other	20	3	15%	0	0%	0	0%	18	90%
Total	249 ^a^	25	10%	19	8%	11	4%	193	78%

UPS: undifferentiated pleomorphic sarcoma; DDLPS: dedifferentiated liposarcoma; SFT: solitary fibrous tumour; MPNST: malignant peripheral nerve sheath tumour. ^a^ 81 of 82 specimens were analysed for SSX2 expression; HPF: high power field.

**Table 3:** Multivariate analysis of overall survival: a Cox proportional hazards model for overall survival was calculated including cancer testis antigens (CTAs) and clinical parameters in a stepwise procedure. HR, hazard ratio.

		HR (95% CI)	*p*
Metastatic disease	M0	1	<0.001
	M1	3.182 (1.875–5.401)	
Surgical margins	R0/R1	1	<0.001
	R2 or no resection	2.531 (1.682–3.809)	
PRAME	Low	1	<0.001
	High	2.675 (1.548–4.622)	
SSX2	High	1	0.039

**Table 4:** Antibodies used for immunohistochemistry staining. ER2: epitope retrieval solution 2.

Antigen	Product No.	Supplier	Clone	Dilution	Pre-Treatment
NY-ESO-1	SC-53869	Santa Cruz	E978	1:100	ER2
PRAME	ab219650	Abcam	EPR20330	1:1000	ER2
SSX2	AMAb91141	Atlas Antibodies	CL3202	1:3000	ER2

## Appendix A

**Table A1:** Frequency of levels of expression for different Cancer Testis Antigens: 0, negative; 1, weak; 2, moderate and 3, strongly positive.

Expression	PRAME		NY-ESO-1		SSX2	
	*n*	%	*n*	%	*n*	%
0	224	90.0	214	85.9	237	95.6
1	6	2.4	8	3.2	2	0.8
2	8	3.2	8	3.2	4	1.6
3	11	4.4	19	7.6	5	2.0
Total	249	100.0	249	100.0	248	100.0
Missing					1	

**Table A2:** Univariate analysis of risk factors for overall survival by Cox regression: HR, hazard ratio.

		HR (95% CI)	*p*
Metastatic disease	M0	1	<0.001
	M1	3.173 (1.889–5.330)	
Surgical margins	R0/R1	1	<0.001
	R2 or no resection	1.618 (1.328–1.972)	
PRAME	Low	1	0.117
	High	1.181 (0.959–1.453)	
SSX2	High	1	0.229
	Low	1.841 (0.681–4.982)	
NY-ESO-1	High	1	0.096
	Low	1.600 (0.921–2.782)	
TILs count	Low	1	0.635
	High	1.207 (0.804–1.811)	
Histological subgroup		1.061 (1.007–1.119)	0.028
Radiotherapy	Not done	1	0.455
	Done	1.166 (0.779–1.743)	
Regional hyperthermia	Not done	1	0.085
	Done	1.446 (0.950–2.200)	
